# Supplementary material for: Systemic Anti-Cancer Therapy and Metastatic Cancer Are Independent Mortality Risk Factors during Two UK Waves of the COVID-19 Pandemic at University College London Hospital
Source: Cancers (Basel). 2021 Dec 2;13(23):6085. doi: 10.3390/cancers13236085 (PMC8657102; doi:10.3390/cancers13236085)
Supplement: Supplementary file 1 [file cancers-13-06085-s001.zip › cancers-1456913-supplementary.pdf]

Supplementary Information

Supplementary Table S1: Comparison of cancer-specific characteristics in wave 1 and 2. Data is shown as n (%). P-value significance level is indicated for the combined cancer cohort in each wave.

	Wave 1 (n = 94)	Wave 2 (n = 113)	*p*-value
**Primary site**			
**Gastrointestinal**	23 (24.5%)	29 (25.7%)	0.84
**Gynaecological**	9 (9.6%)	11 (9.7%)	0.97
**Genitourinary**	24 (25.5%)	27 (23.9%)	0.79
**Thoracic**	15 (16.0%)	13 (11.5%)	0.35
**Breast**	8 (8.5%)	14 (12.4%)	0.37
**Other**	15 (16.0%)	19 (16.8%)	0.86
**Active malignancy**	58 (61.7%)	70 (61.9%)	0.84
**Remission**	50 (53.2%)	41 (36.3%)	
**Metastatic malignancy**	19 (20.2%)	28 (24.8%)	0.42
**SACT**	25 (26.6%)	33 (29.2%)	0.72
**Cytotoxic chemotherapy**	16 (17.0%)	24 (21.2%)	0.44
**Palliative chemotherapy**	9 (9.6%)	16 (14.2%)	0.31
**Immunotherapy**	4 (4.3%)	1 (0.9%)	0.11
**Targeted therapy**	1 (1.1%)	7 (6.2%)	0.06
**Endocrine therapy**	8 (8.5%)	7 (6.2%)	0.51
**Surgery**	3 (3.2%)	5 (4.4%)	0.66

Supplementary Table S2: Univariate analysis of general and cancer-specific variables among patients with cancer. Table shows the hazard ratios (HR) from univariate analyses of risk factors associated with mortality in COVID-19. 95% confidence interval for each HR is shown alongside p-value.

Title	HR (95% CI for HR)	*p*-value
**Age (every 10 years)**	1.41 (1.15-1.73)	<0.001
**Male sex**	1.51 (0.86-2.66)	0.15
**South Asian**	1.79 (0.9-3.56)	0.10
**Black**	0.5 (0.15-1.59)	0.24
**White**	0.73 (0.43-1.25)	0.25
**Other**	1.54 (0.75-3.14)	0.24
**BMI**	0.98 (0.94-1.03)	0.43
**Ex-or-active smoker**	1.07 (0.63-1.84)	0.80

**Dementia**	1 (0.4-2.5)	1.00
**Diabetes**	1.01 (0.55-1.84)	0.98
**Immunosuppression**	3.17 (0.77-13.13)	0.11
**Congestive cardiac failure**	0.9 (0.33-2.49)	0.84
**Liver impairment**	0.85 (0.12-6.13)	0.87
**Hypertension**	1.55 (0.92-2.59)	0.10
**Peripheral vascular disease**	1.05 (0.25-4.29)	0.95
**Stroke**	1.58 (0.8-3.14)	0.19
**Chronic lung disease**	1.1 (0.56-2.18)	0.78
**Chronic kidney disease**	1.24 (0.61-2.52)	0.56
**Steroid therapy in progress**	1.08 (0.26-4.42)	0.92
**Cardiovascular disease**	1.32 (0.73-2.38)	0.35
**Other co-morbidity**	0.8 (0.45-1.4)	0.43
**Wave 2**	0.41 (0.23-0.71)	<0.01
**Gastrointestinal**	1.16 (0.67-2.03)	0.60
**Gynaecological**	1.01 (0.36-2.78)	0.99
**Genito-urinary**	1.34 (0.77-2.34)	0.31
**Thoracic**	0.97 (0.46-2.05)	0.94
**Breast**	0.66 (0.24-1.84)	0.43
**Other**	0.61 (0.26-1.42)	0.25
**Adenocarcinoma**	1.03 (0.6-1.76)	0.92
**Squamous**	0.83 (0.41-1.71)	0.62
**Other**	1.11 (0.61-1.99)	0.74
**Metastatic disease**	1.61 (0.91-2.83)	0.10
**Any anti-cancer therapy**	1.11 (0.64-1.95)	0.71
**Systemic anti-cancer therapy**	1.44 (0.82-2.53)	0.20
**Cytotoxic chemotherapy**	1.17 (0.6-2.26)	0.64
**Neo/adjuvant chemotherapy**	0.57 (0.14-2.34)	0.43
**Palliative chemotherapy**	1.54 (0.75-3.13)	0.24
**Immunotherapy**	1.45 (0.35-5.94)	0.61
**Targeted therapy**	0.61 (0.08-4.41)	0.62
**Endocrine therapy**	1.64 (0.71-3.83)	0.25
**Radiotherapy**	1.46 (0.46-4.69)	0.52
**Surgery**	0.31 (0.04-2.28)	0.25
